# Global incidence of intrahepatic cholestasis of pregnancy: A protocol for systematic review and meta‐analysis

**DOI:** 10.1002/hsr2.1901

**Published:** 2024-02-15

**Authors:** Alireza Sadeghi

**Affiliations:** ^1^ Student Research Committee Shiraz University of Medical Sciences Shiraz Iran

**Keywords:** intrahepatic cholestasis, meta‐analysis, pregnancy, protocol, systematic review

## Abstract

**Background:**

Intrahepatic cholestasis of pregnancy (ICP) typically develops in the late second and third trimesters and resolves rapidly after delivery. Although not associated with serious maternal sequelae, ICP can be highly bothersome. On the other hand, the major complications of ICP are fetal and neonatal, which can be fatal. The current knowledge lacks an estimation regarding the global prevalence of ICP.

**Methods:**

PubMed, Scopus, and Web of Science will be searched systematically. Records will be screened for eligibility by two independent reviewers. Observational studies that reported the incidence of ICP will be eligible. Joanna Bridge Institute checklist for appraisal of prevalence studies will be used for quality assessment. Freeman‐Tukey double arcsine transformed effect sizes will be pooled under random‐effect models. The residual between study heterogeneity will be quantified using *I* ^2^ statistic. Further investigations will be done using subgroup and meta‐regression analyses.

**Discussion:**

Estimating the global and regional prevalence of ICP and evaluating the effects of moderating factors will provide valuable insights into the knowledge. Further investigations on the moderating factors will help researchers to hypothesize the associations and extend the current understanding of the disease. The planned study will be the first systematic review and meta‐analysis that estimates the global prevalence of ICP. The reviewers will try rigorous mythology to ensure high‐quality evidence. However, substantial heterogeneity is expected as prevalence studies utilize different mythologies, settings, and definitions. Moreover, this study relies on utilizing previously published studies, which may impede the overall data quality and comprehensiveness.

## 1 INTRODUCTION

Intrahepatic cholestasis of pregnancy (ICP) is a condition that commonly arises during the later stages of the second and third trimesters, and it tends to alleviate swiftly following delivery. Symptoms of ICP include pruritus and impaired serum bile acid and liver function tests. The underlying mechanisms of ICP are still not fully understood. ICP is associated with adverse obstetrical outcomes such as respiratory distress syndrome, intrauterine demise, fetal asphyxia, and meconium‐stained amniotic fluid. Additionally, ICP can cause significant maternal itching that may be bothersome and unbearable.^1^

ICP is widely recognized as the most prevalent liver disease related to pregnancy.^2^ Its occurrence, however, exhibits variations in geographical, ethnic, and temporal factors.^1^ Previous studies have revealed a higher prevalence of ICP in South America and northern Europe. ICP has been described in 0.2% to 0.3% of pregnancies in the United States.^3^ Additionally, multifetal pregnancies and women of advanced maternal age are more likely to experience this condition.^4^, ^5^ Two studies found that first‐degree relatives of women with a history of ICP had suffered from some kind of liver dysfunction during their pregnancies.^6^, ^7^ However, the current literature lacks high‐quality evidence regarding the global incidence of ICP and its geographical, temporal, and ethnic trends.

At present, a lack of systematic review and meta‐analysis exists regarding the incidence of ICP. Additionally, the current understanding of geographical and ethnical distribution trends relies on disparate original studies, lacking statistical summarization. Conducting a rigorous meta‐analysis would substantially contribute to providing more precise and reliable estimates in this regard. The results of this study have the potential to contribute to the advancement of knowledge of ICP by assisting researchers in generating hypotheses. Furthermore, health policymakers can utilize these findings to support the development of regional diagnostic and screening practices. To ensure the methodological rigor and dependability of the systematic review and meta‐analysis findings, a prior peer‐reviewed protocol will be implemented.

## 2 REVIEW QUESTION

The reviewers aim to achieve a global estimate of the prevalence of ICP. Additionally, this study aims to examine the geographical, temporal, and sociodemographic trends in ICP prevalence by analyzing a compilation of studies conducted in different regions over the past few decades.

## 3 METHODS

This study protocol follows the guidelines set by the Preferred Reporting Items for Systematic Reviews and Meta‐Analysis protocols (PRISMA‐P) 2015.^8^ This protocol is also prospectively registered to PROSPERO (registration ID: CRD42023467481).

### 3.1 Data sources and search strategy

**Figure 1:**
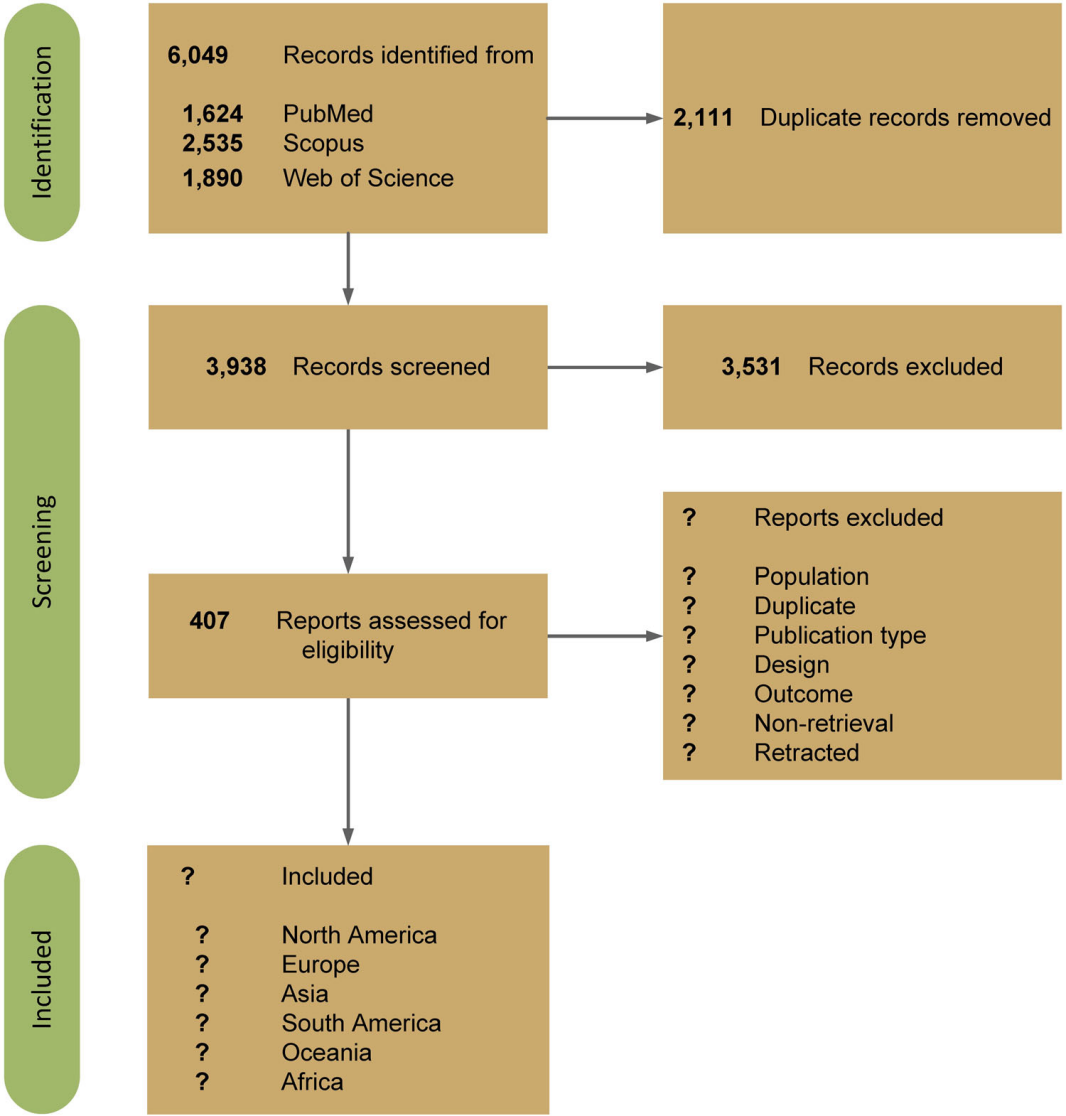
Preferred Reporting Items for Systematic Reviews and Meta‐Analysis (PRISMA) flow diagram demonstrating the current status of the review and primary results.

A systematic search was conducted in PubMed, Scopus, and Web of Science online databases for records published since inception until July 13th, 2023. A search strategy of combination of “intrahepatic cholestasis,” “pregnancy,” and related keywords was used to gather all the potential records (provided in Supporting Information 1). Figure 1 presents the PRISAM flow diagram of the current review status.

### 3.2 Screening for eligibility

Two independent reviewers will screen the records based on titles and abstracts using the Rayyan online tool for managing systematic reviews.^9^ The records that pass the first round of screening will then be checked for eligibility based on their full texts. Discussions will be made to resolve conflicts. If not resolved, a third reviewer with more experience will make the final decision. When providing insufficient data, the reviewers will make contact with the authors through their publicly available email addresses to gather the information needed for analyses. If no answers were received over a 1‐month period, the studies will be removed from the review.

Published and preprint observational reports (cohort, cross‐sectional, and case‐control), posters, and conference abstracts are eligible for inclusion if sufficient data (the prevalence of ICP) were provided.^10^ No time and language restrictions are applicable to reduce language and publication bias.^11^

### 3.3 Data extraction

Data will be extracted in duplicate and entered into separate Excel spreadsheets. Two independent reviewers will meticulously verify the extracted data to identify and rectify any errors. Study‐level characteristics, including first author, publication year, publication type, study year(s), study country, sample size (gestating females), number of gravid women with ICP, mean age, mean body mass index, diagnostic criteria for ICP, season(s) the data were gathered, parity of the included mothers, and singleton/multitone pregnancies will be extracted from the included studies. Also, more potential moderating covariates and factors will be decided to add during data extraction. In case of challenging data structures, the approach that will be devised during quantitative analysis and reporting will follow our previous publication in meta‐analysis of single proportion.^12^

### 3.4 Quality assessment

The Joanna Bridge Institute (JBI) checklist for prevalence studies will be employed by two independent reviewers to evaluate the quality of the studies included.^13^, ^14^ Disagreements will be effectively resolved through thorough discussions and the active participation of a third reviewer. The checklist of JBI critical appraisal tool for prevalence studies is available in Supporting Information 2.

### 3.5 Analyses

Stata version 16 (StataCorp) will be utilized for meta‐analysis. DerSimonian‐Laird random effects model will be used to pool the Freeman‐Tukey double arcsine transformed effect sizes. The Freeman‐Tukey transformation method will reduce the effect of extremely high or low data on the pooled estimates.^15^, ^16^ Cochrane's *Q* statistic, *I* ^*2*^, and *p* value will quantify the residual between‐study heterogeneity. *I* ^*2*^ of more than 75% will be considered high.^17^ Forest plots will be employed for visual representation of the results.

Possible sources of residual between‐study heterogeneity will be explored using subgroup and meta‐regression analyses. Major moderating covariates and factors will be mean age, mean body mass index, geographical longitude and latitude, world health classification, world bank income grouping, and global hemisphere of the country the study was published from, major components of JBI appraisal tool (adequacy of the sample size and validation of the identification method), seasonal variations, temporal trends, and the peer‐review status of the records. The results of subgroup analyses will be visually represented using forest plots and Voronoi treemaps.^18^

The sensitivity of the final results will be measured using the leave‐one‐out method and subgroup analyses based on quality assessments. Publication bias will not be assessed in this review, as it is generally discouraged for meta‐analyses of prevalence studies.^19^

## 4 DISCUSSION

Previous publications have provided estimates of the global incidence of ICP ranging from 0.2% to 2% of pregnancies. However, due to the lack of a systematic review and meta‐analysis, the true global incidence of ICP remains uncertain. Thus, our planned systematic review and meta‐analysis aims to fill this gap by comprehensively gathering evidence and providing a statistically robust estimation of ICP incidence. Additionally, we intend to explore geographical, temporal, ethnic, and sociodemographic trends through further investigations. This study will thereby serve as the first comprehensive assessment of ICP incidence and its associated factors at a global level.

Insufficient evidence in healthcare practices can lead to serious consequences for the public health economy.^20^ Therefore, it is crucial to generate comprehensive results that are of interest to researchers, clinicians, and policymakers alike. Researchers can leverage subgroup and meta‐regression analyses to formulate hypotheses about associations and guide future studies. Additionally, clinicians and policymakers can utilize information about regional prevalence to strategically plan their diagnostic practices.

The planned systematic review and meta‐analysis represent a significant contribution to the field as it will be the first study to estimate the incidence of ICP through this rigorous methodology. The inclusion of a comprehensive search strategy and inclusive eligibility criteria will ensure a larger pool of relevant studies, resulting in improved analysis power and more representative, reliable, and accurate findings. By not limiting the inclusion criteria to peer‐reviewed and English studies, the study will minimize publication bias. Moreover, the inclusion of extensive subgroup and meta‐regression analyses will provide high‐quality evidence that addresses global and regional demands, allowing for a comprehensive exploration of regional, temporal, and ethical trends associated with ICP.

However, it is important to recognize the limitations of this study. Meta‐analyses of prevalence commonly show high heterogeneity due to variations in methodology.^21^, ^22^, ^23^, ^24^ The sources of this heterogeneity will be further explored. Additionally, since this review will include previously published studies, the reliability and certainty of the evidence will depend on the quality of the data reported in those studies. Furthermore, it is expected that the published studies will primarily come from a limited number of countries, as some countries are less active in publishing scientific reports. Therefore, caution should be exercised when interpreting the conclusions drawn from these findings.

### 4.1 Current status

The reviewers conducted a thorough search of relevant databases and assessed the titles and abstracts of the retrieved records, resulting in a total of 407 studies. To create a structured data extraction form, a preliminary data extraction of 10 studies was undertaken. Once this protocol is approved, further progress will be made.

### 4.2 Reporting of this review

The proposed systematic review will be reported following the PRISMA guidelines. We intend to publish a PRISMA checklist alongside the final report.

### 4.3 Potential amendments

Although amendments are not intended, inevitable changes to the review will be reported transparently.

## AUTHOR CONTRIBUTIONS

**Alireza Sadeghi**: Conceptualization; Investigation; Methodology; Resources; Writing—original draft; Writing—review & editing.

## CONFLICT OF INTEREST STATEMENT

The author declares no conflict of interest.

## TRANSPARENCY STATEMENT

The lead author Alireza Sadeghi affirms that this manuscript is an honest, accurate, and transparent account of the study being reported; that no important aspects of the study have been omitted; and that any discrepancies from the study as planned (and, if relevant, registered) have been explained.

## Supporting information


**Supporting Information 1**: Search strategy.

**Supporting Information 2:** JBI checklist for critical appraisal of prevalence studies. JBI: Joanna Bridge Institute.

## Data Availability

Data sharing is not applicable to this article, as no data sets were generated or analyzed during the current study.
