# Supplementary material for: Global incidence of intrahepatic cholestasis of pregnancy: A protocol for systematic review and meta‐analysis
Source: Health Sci Rep. 2024 Feb 15;7(2):e1901. doi: 10.1002/hsr2.1901 (PMC10867694; doi:10.1002/hsr2.1901)
Supplement: Supplementary file 1 — Supporting Information 1: Search strategy. [file HSR2-7-e1901-s001.docx]

# PubMed

("pregnancy"[Title/Abstract] OR "pregnant"[Title/Abstract] OR "gestational"[Title/Abstract] OR "pregnancy"[MeSH Terms]) AND ("cholestasis"[Title/Abstract] OR "cholestatic"[Title/Abstract] OR (("biliary"[Title/Abstract] OR "hepatobiliary"[Title/Abstract] OR "bile duct"[Title/Abstract]) AND ("obstruction"[Title/Abstract] OR "stasis"[Title/Abstract])) OR "cholestasis, intrahepatic"[MeSH Terms]) AND ("intrahepatic"[Title/Abstract] OR "intra-hepatic"[Title/Abstract])

Results: 1,624

Date: July 13th, 2023

# Scopus

TITLE-ABS-KEY ( "pregnancy" OR "pregnant" OR "gestational" ) AND TITLE-ABS-KEY ( "cholestasis" OR "cholestatic" OR ( ( "biliary" OR "hepatobiliary" OR "bile duct" ) AND ( "obstruction" OR "stasis" ) ) ) AND TITLE-ABS-KEY ( "intrahepatic" OR "intra-hepatic" )

Results: 2,535

Date: July 13th, 2023

# Web of Science

TS=("pregnancy" OR "pregnant" OR "gestational") AND TS=("cholestasis" OR "cholestatic" OR (("biliary" OR "hepatobiliary" OR "bile duct") AND ("obstruction" OR "stasis"))) AND TS=("intrahepatic" OR "intra-hepatic")

Results: 1,890

Date: July 13th, 2023

**Duplicates: 2,111**
