# Supplementary figures and images for: Global incidence of intrahepatic cholestasis of pregnancy: A protocol for systematic review and meta‐analysis
Source: Health Sci Rep. 2024 Feb 15;7(2):e1901. doi: 10.1002/hsr2.1901 (PMC10867694; doi:10.1002/hsr2.1901)

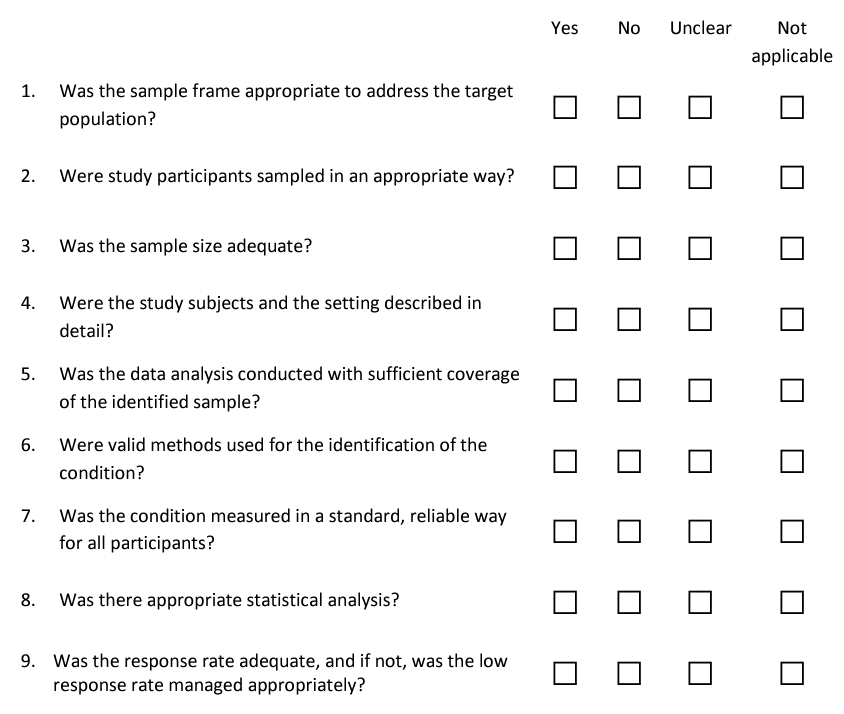

Supplement: Supplementary file 2 — Supporting Information 2: JBI checklist for critical appraisal of prevalence studies. JBI: Joanna Bridge Institute. [file HSR2-7-e1901-s002.png]
